# Supplementary material for: Estimation of Minced Pork Microbiological Spoilage through Fourier Transform Infrared and Visible Spectroscopy and Multispectral Vision Technology
Source: Foods. 2019 Jul 1;8(7):238. doi: 10.3390/foods8070238 (PMC6678698; doi:10.3390/foods8070238)
Supplement: Supplementary file 1 [file foods-08-00238-s001.pdf]

Supplementary Materials: The following are available online at www.mdpi.com/xxx/s1, TableS1: Estimated growth kinetic parameters of total mesophiles (total viable counts, TVC), *Pseudomonas* spp., lactic acid bacteria (LAB) and *Brochothrix thermosphacta* in minced pork samples stored aerobically at 4, 8, 12 °C and at dynamic temperature conditions.

Table S1. Estimated growth kinetic parameters of total mesophiles (total viable counts, TVC), *Pseudomonas* spp., lactic acid bacteria (LAB) and *Brochothrix thermosphacta* in minced pork samples stored aerobically at 4, 8, 12 °C and at dynamic temperature conditions.

Microbial group	Temperature	μ_{max} (h^{-1})*	λ (h)*	y_0 (log CFU/g)*	y_{end} (log CFU/g)*	Standard error of fitting (minimum-maximum)	R^2 (minimum-maximum)
TVC	4 °C	0.079 ± 0.018	33.23 ± 6.82	3.47 ± 0.28	9.52 ± 0.16	0.199-0.381	0.971-0.993
Pseudomonas spp.		0.084 ± 0.010	29.99 ± 9.58	2.32 ± 0.46	9.34 ± 0.42	0.179-0.424	0.972-0.995
LAB		0.051 ± 0.007	28.55 ± 13.08	2.47 ± 0.23	6.96 ± 0.34	0.314-0.404	0.940-0.962
Brochothrix thermosphacta		0.083 ± 0.007	18.60 ± 10.36	1.94 ± 0.02	8.05 ± 0.20	0.178-0.326	0.981-0.994
TVC	8 °C	0.127 ± 0.012	18.20 ± 6.73	3.41 ± 0.38	9.38 ± 0.32	0.190-0.330	0.980-0.993
Pseudomonas spp.		0.146 ± 0.013	21.94 ± 13.07	2.34 ± 0.57	9.28 ± 0.39	0.279-0.362	0.981-0.990
LAB		0.115 ± 0.010	17.52 ± 2.067	2.39 ± 0.18	7.33 ± 0.25	8221-0.555	0.911-0.987
Brochothrix thermosphacta		0.141 ± 0.005	16.19 ± 4.63	1.93 ± 0.06	7.87 ± 0.21	0.230-0.326	0.980-0.990
TVC	12 °C	0.222 ± 0.009	16.10 ± 4.46	3.37 ± 0.28	9.12 ± 0.24	0.265-0.363	0.975-0.988

Pseudomonas spp.		0.212 ± 0.026	17.08 ± 9.00	2.33 ± 0.40	9.07 ± 0.24	0.226-0.550	0.961-0.991
LAB		0.193 ± 0.053	11.95 ± 9.87	2.62 ± 0.14	7.57 ± 0.31	0.242-0.450	0.947-0.985
Brochothrix thermosphacta		0.201 ± 0.008	8.85 ± 2.65	1.89 ± 0.07	7.70 ± 0.29	0.155-0.269	0.985-0.995
TVC	Dynamic	0.131 ± 0.010	17.33 ± 3.64	3.25 ± 0.35	9.30 ± 0.37	0.288-0.373	0.976-0.985
Pseudomonas spp.		0.154 ± 0.021	22.70 ± 12.25	2.39 ± 0.54	9.11 ± 0.32	0.277-0.348	0.981-0.990
LAB		0.124 ± 0.009	22.93 ± 4.63	2.41 ± 0.14	7.45 ± 0.26	0.206-0.326	0.973-0.990
Brochothrix thermosphacta		0.158 ± 0.017	20.73 ± 6.71	1.87 ± 0.06	7.88 ± 0.18	0.240-0.340	0.978-0.990

* Values are means ± standard deviations (*n*=4) of the growth kinetic parameters estimated by the Baranyi model [17]. μ_{max}: maximum specific growth rate; λ: lag time; y_0: initial microbial population; y_{end}: maximum population density.
